# 1639. A Review of Clinical Safety, Reactogenicity, and Immunogenicity of an mRNA-Based Seasonal Influenza Vaccine (mRNA-1010) in Adults

**DOI:** 10.1093/ofid/ofad500.1473

**Published:** 2023-11-27

**Authors:** Mieke Soens, Boris Kandinov, Daniela Ramirez-Schrempp, Jignesh Vakil, Alicia Pucci, Wenmei Huang, Jia Guo, Evelyn Du, Andrei Avanesov, Sinead Rudden, Bryony Hicks, Greg Livermore, Kristi Schaefers, Mary Lau, Raffael Nachbagauer, Jintanat Ananworanich

**Affiliations:** Moderna, Inc, Cambridge, Massachusetts; Moderna, Inc, Cambridge, Massachusetts; Moderna, Inc, Cambridge, Massachusetts; Moderna, Inc, Cambridge, Massachusetts; Moderna, Inc, Cambridge, Massachusetts; Moderna, Inc, Cambridge, Massachusetts; Moderna, Inc, Cambridge, Massachusetts; Moderna, Inc, Cambridge, Massachusetts; Moderna, Inc, Cambridge, Massachusetts; Moderna, Inc, Cambridge, Massachusetts; Moderna, Inc, Cambridge, Massachusetts; Moderna, Inc, Cambridge, Massachusetts; Moderna, Inc, Cambridge, Massachusetts; Moderna, Inc, Cambridge, Massachusetts; Moderna, Inc, Cambridge, Massachusetts; Moderna, Inc, Cambridge, Massachusetts

## Abstract

**Background:**

Seasonal influenza remains a public health threat with approximately 1 billion global infections annually. Vaccines based on messenger RNA (mRNA) technology are currently under investigation and have the potential to address limitations of current seasonal influenza vaccine platforms. Here, we review recent phase 1-3 randomized clinical trials evaluating an mRNA-based, quadrivalent seasonal influenza vaccine (mRNA-1010) in adults.

**Methods:**

Safety, reactogenicity, and immunogenicity of a single dose of mRNA-1010 were assessed in adults aged ≥18 years in phase 1/2 (NCT04956575) and ongoing phase 3 immunogenicity (NCT05415462) trials and aged ≥50 years in an ongoing phase 3 efficacy (NCT05566639) trial. Safety endpoints were solicited local and systemic adverse reactions (ARs) up to 7 days after vaccination, unsolicited adverse events (AEs) up to 28 days after vaccination, and serious AEs (SAEs), AEs of special interest (AESIs), medically attended AEs (MAAEs), and AEs leading to study discontinuation. Immunogenicity was measured by hemagglutination inhibition assay. Available safety and immunogenicity data up to 6 months (phase 1/2) and up to 3 months (phase 3) after vaccination are summarized here.

**Results:**

Approximately 14,400 participants received mRNA-1010 at the 50-µg dose level across trials. No safety concerns for mRNA-1010 have been identified for any trial. Overall, pain and axillary swelling/tenderness were the most common solicited local ARs while myalgia, headache, and fatigue were the most common systemic ARs. In the phase 1/2 and phase 3 immunogenicity trials, solicited ARs were more frequent after vaccination with mRNA-1010 than after an active comparator (licensed quadrivalent seasonal influenza vaccine). No vaccine-related SAEs were observed through 6 months after vaccination in the phase 1/2 trial; SAEs, AESIs, and MAAEs up to 3 months after vaccination were similar between mRNA-1010 and an active comparator group in the 2 phase 3 trials. A single dose of mRNA-1010 induced immune responses against influenza A and B strains included in the vaccine.

**Conclusion:**

mRNA-1010 has demonstrated an acceptable safety and tolerability profile in phase 1-3 studies. These data support continued investigation of mRNA-based vaccines against seasonal influenza.

**Disclosures:**

**Mieke Soens, MD**, Moderna, Inc.: Employee|Moderna, Inc.: Stocks/Bonds **Boris Kandinov, MD**, Moderna, Inc.: Employee|Moderna, Inc.: Stocks/Bonds **Daniela Ramirez- Schrempp, MD**, Moderna, Inc.: Employee|Moderna, Inc.: Stocks/Bonds **Jignesh Vakil, MD**, Moderna, Inc.: Employee|Moderna, Inc.: Stocks/Bonds **Alicia Pucci, BSN**, Moderna, Inc.: Employee|Moderna, Inc.: Stocks/Bonds **Wenmei Huang, PhD**, Moderna, Inc.: Employee|Moderna, Inc.: Stocks/Bonds **Jia Guo, PhD**, Moderna, Inc.: Employee|Moderna, Inc.: Stocks/Bonds **Evelyn Du, PhD**, Moderna, Inc.: Employee|Moderna, Inc.: Stocks/Bonds **Andrei Avanesov, PhD**, Moderna, Inc.: Employee|Moderna, Inc.: Stocks/Bonds **Sinead Rudden, PhD**, Moderna, Inc.: Employee|Moderna, Inc.: Stocks/Bonds **Bryony Hicks, BSc**, Moderna, Inc.: Employee|Moderna, Inc.: Stocks/Bonds **Greg Livermore, BS, MBA**, Moderna, Inc.: Employee|Moderna, Inc.: Stocks/Bonds **Kristi Schaefers, BIS**, Moderna, Inc.: Employee|Moderna, Inc.: Stocks/Bonds **Mary Lau, BS**, Moderna, Inc.: Employee|Moderna, Inc.: Stocks/Bonds **Raffael Nachbagauer, MD, PhD**, Moderna, Inc.: Employee|Moderna, Inc.: Stocks/Bonds **Jintanat Ananworanich, MD, PhD**, Moderna, Inc.: Employee|Moderna, Inc.: Stocks/Bonds

